# Disruption of Microtubule Network Rescues Aberrant Actin Comets in Dynamin2-Depleted Cells

**DOI:** 10.1371/journal.pone.0028603

**Published:** 2011-12-12

**Authors:** Yuji Henmi, Kenji Tanabe, Kohji Takei

**Affiliations:** Department of Neuroscience, Graduate School of Medicine, Okayama University Okayama, Japan; Bernhard Nocht Institute for Tropical Medicine, Germany

## Abstract

A large GTPase dynamin, which is required for endocytic vesicle formation, regulates the actin cytoskeleton through its interaction with cortactin. Dynamin2 mutants impair the formation of actin comets, which are induced by *Listeria monocytogenes* or phosphatidylinositol-4-phosphate 5-kinase. However, the role of dynamin2 in the regulation of the actin comet is still unclear. Here we show that aberrant actin comets in dynamin2-depleted cells were rescued by disrupting of microtubule networks. Depletion of dynamin2, but not cortactin, significantly reduced the length and the speed of actin comets induced by *Listeria*. This implies that dynamin2 may regulate the actin comet in a cortactin-independent manner. As dynamin regulates microtubules, we investigated whether perturbation of microtubules would rescue actin comet formation in dynamin2-depleted cells. Treatment with taxol or colchicine created a microtubule-free space in the cytoplasm, and made no difference between control and dynamin2 siRNA cells. This suggests that the alteration of microtubules by dynamin2 depletion reduced the length and the speed of the actin comet.

## Introduction

Dynamin GTPase plays an essential role in vesicle formation during endocytosis [1]. Dynamin oligomerizes around the neck of the clathrin coat pit, and its GTPase activity is required for fission of the constricted membrane to produce endocytic vesicles. Dynamin has three isoforms in mammals. Dynamin1 is specifically expressed in the brain, dynamin2 is ubiquitously expressed, and dynamin3 is expressed in neurons and testes [2], [3]. All dynamin isoforms have five domains—the N-terminal GTPase domain, middle domain, pleckstrin homology domain, GTPase effector domain, and C-terminal proline rich domain (PRD). The GTPase domain plays a role in the hydrolysis of GTP. PRD connects with other SH3 domain-containing proteins, such as amphiphysin, intersectin, and cortactin [4], [5].

Recently, it has been suggested that dynamin interacts with cortactin to regulate actin assembly [6]. Cortactin binds F-actin and induces an actin meshwork by activating the Arp2/3 complex [7]. The interaction between dynamin and cortactin plays a key role in the membrane deformation involved in cell motility, endocytic vesicle formation, and propulsive force [8], [9], [10], [11], [12], [13].

Dynamin was originally identified as a specific microtubule-binding GTPase [14], [15], [16], [17]. Recent research has shown that a dynamin2 mutant, which was found in a neuropathy, induces the accumulation of stable microtubules [18]. Thus, dynamin has multiple functions other than endocytic fission.

Dynamin2 and cortactin localize in the actin comet induced by *Listeria* or PIP5K overexpression, and dynamin2 mutants (K44A, D208N, and ΔPRD) perturb actin comet formation [19], [20]. However, another study showed that cortactin is not essential for actin comet formation [21].

Several bacteria (*Listeria* and *Shigella*) and viruses recruit and activate the Arp2/3 complex in host cells to produce an actin comet [22]. After entering the host cell, *Listeria* can escape from the endosome using listeriolysin O. Once in the cytosol, *Listeria* induces nucleation and assembly of the host cell actin filaments. This can generate substantial force, pushing these pathogens forward through the cytoplasm [23]. To induce actin nucleation, *Listeria* expresses a surface protein, ActA, which directly binds to and activates the ARP2/3 complex. After escaping from the endosome, *Listeria* is surrounded by actin (actin cloud). Then, an actin comet is created on the side of the bacterium, producing motile force that moves the bacterium rapidly through the host cell. This allows the spread of bacteria from cell to cell [23]. Although dynamin2 was found in the actin comet, its significance remains to be elucidated.

In this study, we investigated the role of endogenous dynamin2 in the regulation of the actin comet. Dynamin2 depletion, but not cortactin, reduced the length and the speed of actin comets. We found that microtubules are essential to the differences between control and dynamin2-depleted cells. These results indicate that the alteration (such as increased stability) of microtubules by dynamin2 depletion may be responsible for the reduction of the length of the actin comet.

## Results

### Dynamin2 and cortactin were differentially recruited to actin comet

**Figure 1:**
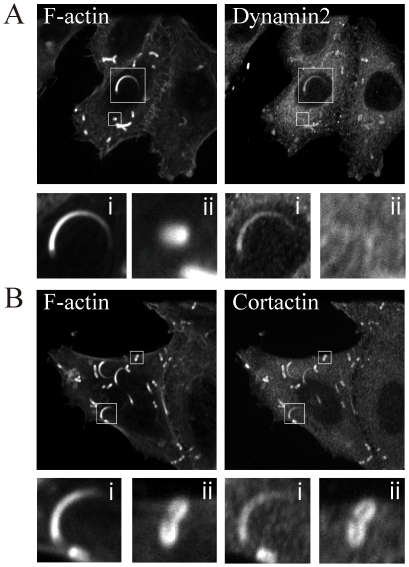
Endogenous dynamin2 and cortactin are present in the actin comet. HeLa cells were infected with *Listeria* and immunolabeled with F-actin, dynamin2, or cortactin. (A) Dynamin2 was detected in the actin comet (arrowhead and inset i) but not the actin cloud (arrow and inset ii). (B) Cortactin was detected in the actin comet (arrowhead and inset i) and actin cloud (arrow and inset ii). The lower panels are enlargements of the boxed area. Bar, 20 µm.

To confirm localization of endogenous dynamin2 and cortactin in the actin comet, HeLa cells were infected with *Listeria* for 1 h and then placed in bacteria-free medium for 5 h before fixing. The HeLa cells were immunolabeled for F-actin and dynamin2 or cortactin. As shown in Fig. 1A, endogenous dynamin2 was detected only in the actin comet tail, but not in actin clouds. In contrast, endogenous cortactin was detected in both actin comet tails and actin clouds (Fig. 1B). Thus, dynamin2 and cortactin were differentially recruited to actin clouds and actin comet tails, suggesting that they may play distinct roles on actin comets.

### Dynamin2 siRNA reduced the length of actin comet tail

**Figure 2:**
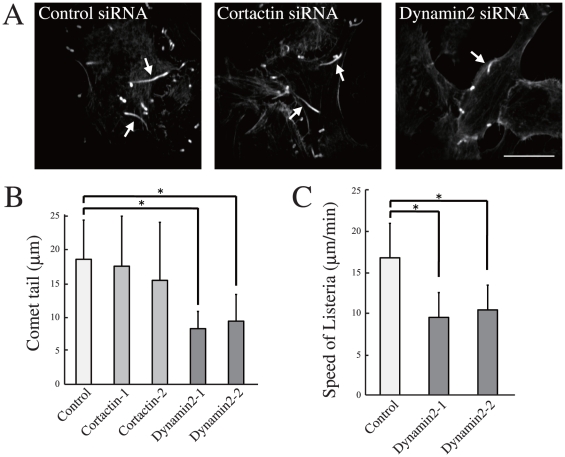
Depletion of dynamin2 reduced the formation of the actin comet. HeLa cells were transfected with control, dynamin2, or cortactin siRNA. (A) At 72 h after transfection, cells were infected with *Listeria*, fixed, and immunolabeled with phalloidin. Arrows indicate actin comets. Bar, 20 µm. (B) Quantitative analysis performed on the results of three independent experiments to measure the length of actin comet. n = 20. (C) HeLa cells stably expressing GFP-actin were transfected with control or dynamin2 siRNA and infected with *Listeria*. Then, *Listeria* motility was observed by time-lapse imaging. *Listeria* movement was measured using ImageJ and represented as mean ± SD of 20 bacteria. *, p<0.005.

Next, we analyzed the role of endogenous dynamin2 and cortactin using siRNA. HeLa cells were transfected with specific siRNAs, infected by *Listeria*, fixed, and processed for immunofluorescence procedures. As shown in Figs. 2A and B, we found that the length of the actin comet tail was reduced in dynamin2 siRNA cells (18.4±5.8 µm in control cells, 8.2±2.5 µm and 9.3±3.9 µm in dynamin2 siRNA cells; n = 20, P<0.001). This was observed with two different dynamin2-specific siRNAs, indicating that the reduction resulted from the lack of dynamin2. On the other hand, cortactin siRNA cells showed no significant reduction in the comet length. These results support those of a previous report, which showed that cortactin does not play any significant role in actin comet formation [21]. These results clearly indicate that dynamin, but not cortactin, plays an indispensable role in actin comet formation.

### Dynamin2 siRNA reduced actin comet formation

We found that dynamin2 siRNA, but not cortactin siRNA, reduced the length of the actin comet tail. To investigate whether the reduction also affected the motility of the intracellular pathogen, we used live cell imaging to observe the dynamics of actin comet formation. Cells stably expressing GFP-actin were transfected with control or dynamin2 siRNA and imaged by time-lapse microscopy after *Listeria* infection. As shown in Movie S1 and S2, the speed of *Listeria* movement was significantly decreased in dynamin2 siRNA cells. Quantitative analysis (Fig. 2C) also showed a significant reduction in the speed of *Listeria* movement (16.7±4.2 µm/min in control cells, 9.5±3.0 µm/min and 10.4±3.0 µm/min in dynamin2 knock down cells; n = 20, P<0.001). These results indicate that dynamin2 depletion reduced both the comet tail length and speed of *Listeria* movement.

### Perturbation of microtubules rescued the effect of dynamin2 siRNA

**Figure 3:**
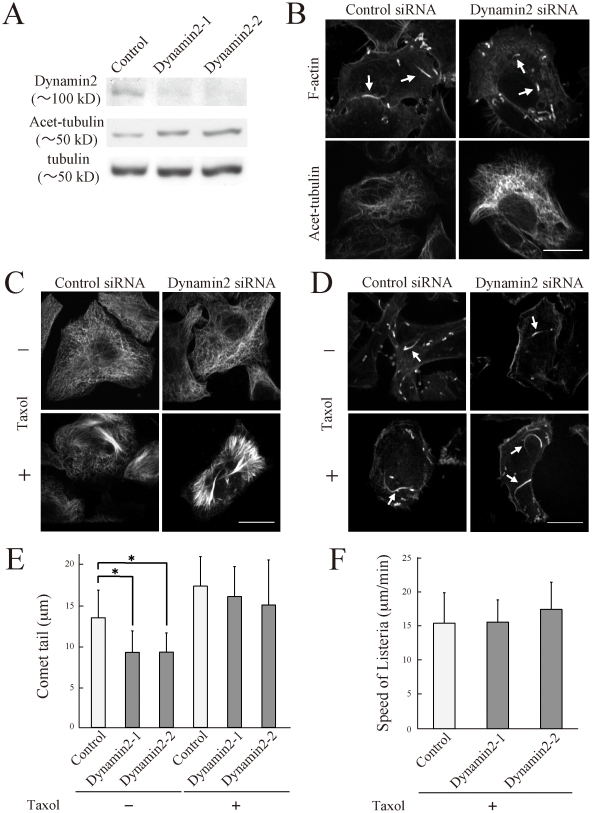
Taxol treatment rescued the reduction of actin comet formation induced by dynamin2 depletion. HeLa cells were transfected with control or dynamin2 siRNA for 72 h and processed for immunoblot analysis or immunofluorescent procedure. (A) Immunoblot analysis was visualized with anti-dynamin2, anti-acetylated tubulin (Acet-tubulin) or anti-α-tubulin. (B) The siRNA transfedted cells were infected with *Listeria* for 1 h, fixed and immunolabeled with phalloidin and anti-acetylated tubulin. Bar, 30 µm. The siRNA transfected cells were infected with *Listeria* for 1 h and placed in medium containing taxol (50 µg/ml; lower panels) for 5 h, fixed and immunolabeled with andti-α-tubulin (C), and phalloidin (D). Arrows indicate actin comet. Bar, 20 µm. (E) The length of 20 actin comets were measured and is presented as the mean ± SD of 20 bacteria. (F) GFP-actin expressing cells were transfected with control or dynamin2 siRNA, infected, and treated with taxol. *Listeria* movement was measured using ImageJ. n = 10. *, p<0.005.

As cortactin depletion had no significant effect on the actin comet, dynamin2 probably regulates the actin comet in a cortactin-independent manner. Microtubules are considered to act as a barrier to the actin comet of bacterial pathogens [24]. We recently found that dynamin2 regulates the dynamic instability of microtubules, and depletion of dynamin2 induces the accumulation of acetylated tubulin, a marker of stable microtubules (Fig. 3A) [18]. Thus, the accumulation of stable microtubules by dynamin2 depletion might form a strong barrier against bacterial pathogens, resulting in the inhibition of actin comet formation (Fig. 3B). To investigate whether microtubule perturbation rescues the reduction in actin comet formation induced by dynamin2 siRNA, we treated *Listeria*-infected cells with microtubule-perturbing reagents. As the microtubule depolymerizing reagent nocodazole dramatically reduces the infectivity of bacteria, we used taxol to create a space for bacteria in the cytoplasm by aggregating microtubules. Infected cells were treated with taxol for 5 h, fixed, and processed for immunofluorescence procedures (Fig. 3C). In taxol-treated cells, microtubules showed abnormal polymerization and microtubule-free spaces were observed. Thus, the taxol treatment appeared to be useful for creating a microtubule-free space.

**Figure 4:**
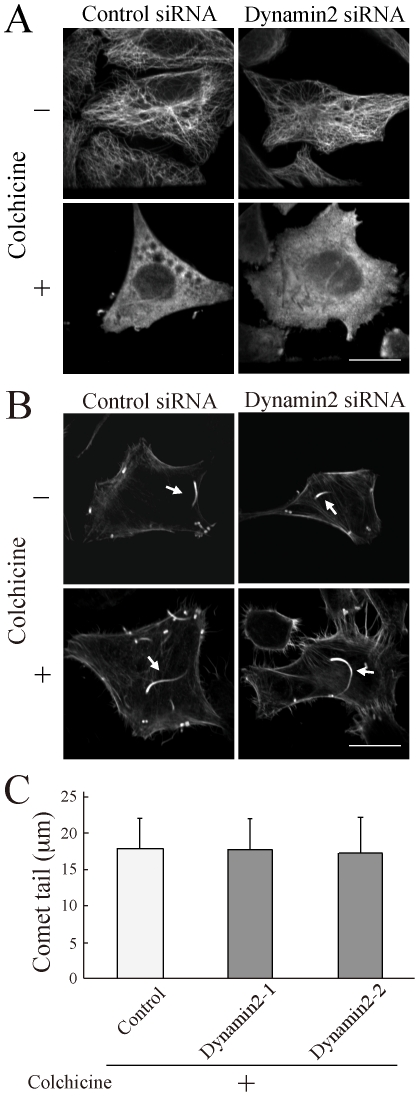
Disruption of microtubule polymerization also rescued the reduction of actin comet formation induced by dynamin2 depletion. HeLa cells were transfected with control or dynamin2 siRNA for 72 h and then infected with *Listeria* for 1 h. Cells were placed in medium containing colchicine (10 µM; lower panels) for 5 h. Cells were fixed and immunolabeled with anti-α-tubulin (A) or phalloidin (B). Bar, 20 µm. Arrows indicate actin comet tails. (C) The length of 10 actin comets was measured and is presented as the mean ± SD of 10 bacteria.

Next, we investigated whether actin comet formation in dynamin2 siRNA cells was affected by the taxol treatment. As shown in Fig. 3D, the length of the comet tail was increased by the taxol treatment in both control and dynamin2 siRNA cells, and no significant differences were observed between these siRNA cells (Fig. 3E). Further, we used live cell imaging of GFP-actin to observe the speed of *Listeria* movement. Consistent with the immunofluorescence experiment, we found that *Listeria* was faster in dynamin2 siRNA cells treated with taxol than in untreated dynamin2 siRNA cells (Movie S1, 2, 3, 4). Quantitative analysis also showed that the speed of *Listeria* movement in dynamin2-depleted cells with taxol was comparable to that in control cells (Fig. 3F). Similar experimetns were performed by using colchicine, a microtubules depolymerizing drug (Fig. 4). Colchicine inhibited microtubule polymerization in both control and dynamin2 siRNA cells (Fig. 4A), and elongated the actin comets in dynamin2 siRNA cells (Fig. 4B and C). Thus, microtubules appear to be responsible for the reduction of the comet tail length in dynamin2 siRNA cells.

**Figure 5:**
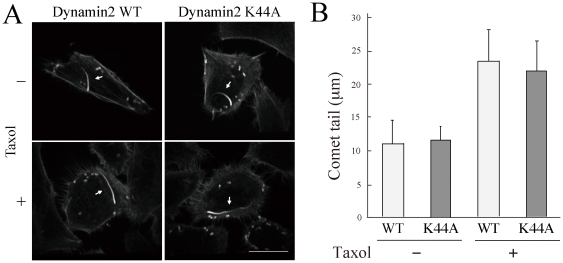
Taxol treatment elongated actin comet in the presence of dynamin2 dominant negative mutant. HeLa cells were transfected with dynamin2 WT-V5/His or dynamin2 K44A-V5/His for 24 h, and infected with *Listeria* for 1 h. Cells were placed in taxol-containing medium (50 µg/ml; lower panels) for 5 h. Cells were fixed and processed for immunofluorescence procedures. The expression of dynamin2 WT or K44A was confirmed by anti-V5 antibody (not shown). (B) The length of actin comets was measured and is presented as the mean ± SD of 10 bacteria. Bar, 20 µm.

Previous studies have shown that the expression of dynamin2 K44A (a dominant-negative mutant) reduced the length and the speed of PIP5K-induced actin comet [19], [20]. As dynamin2 K44A has no effect on the dynamic instability of microtubules, we investigated the effect on *Listeria*-induced actin comet. Interestingly, when dynamin2 K44A was introduced, no significant difference in the actin comet length was observed (Fig. 5A). The mutant effectively inhibited the endocytosis of transferrin (the internalized transferrin was not detected in 0.05% and 93% of WT and K44A-expressing cells, respectively), indicating that the expression level was sufficient to inhibit endogenous dynamin2. Moreover, taxol treatment was also elongated the actin comet even on dynamin2 K44A expressing cells (Fig. 5B).

These results indicate that the reduction of the length and the speed of *Listeria*- induced actin comet in dynamin2-depleted cells could result, at least in part, from the state of microtubules.

## Discussion

Here we investigated the role of dynamin2 and cortactin in actin comets. Both dynamin2 and cortactin localized in the actin comet tail, but cortactin also localized in the actin cloud. These proteins seem to play a distinct role in actin comet formation.

To investigate the role of dynamin2 and cortactin in the actin comet, we depleted endogenous protein using siRNA. Cortactin depletion had no significant effect on the length of the actin comet, as previously reported [21], but dynamin2 depletion significantly reduced both the comet tail length and speed of *Listeria* movement. As dynamin2 regulates actin polymerization via cortactin [9], it is possible that dynamin2 regulates the actin comet in an cortactin/actin-independent manner. A recent finding indicates that dynamin2 directly binds the actin filaments and may regulates its organization [25]. This suggests that dynamin2 may regulate the actin polymerization of the actin comet in cortactin-independent manner. Whereas, dynamin2 regulates the dynamic instability of microtubules, and we investigated in this study whether microtubules are responsible for the aberrant actin comet induced by the depletion of dynamin2.

The cytoplasm of a cell is a dense, organized, tightly regulated structure [24]. Microtubules are considered to act as a barrier to the actin comet. *Shigella* uses the protease VirA, which is secreted by the bacteria itself, to destroy microtubules in host cells, thus facilitating its movement [26]. In the case of *Listeria*, the bacterium recruits stathmin, a microtubule-sequestering protein of the host cell, to destabilize microtubules, thus allowing bacterial movement in the cytoplasm [27]. On the other hand, we have found that dynamin2 depletion increases the stability of microtubules [18]. Therefore, it is possible that the accumulation of stable microtubules, which was induced by dynamin2 siRNA, reduced the comet tail length and speed of *Listeria* movement. Interestingly, no significant difference between control and dynamin2 siRNA cells was observed in taxol-treated cells. Although both dynamin2 depletion and taxol treatment resulted in microtubule stabilization, opposite effect on actin comet formation was acquired. In dynamin2 siRNA cells, dynamic instability of microtubules was reduced, but overall structure of microtubule network was unaffected. On the other hand, taxol prevents only microtubule depolymerization and induces the accumulation of microtubule bundles. This resulted in making a free space for *Listeria* movement in taxol treated cells, but not in dynamin2 siRNA cells.

**Figure 6:**
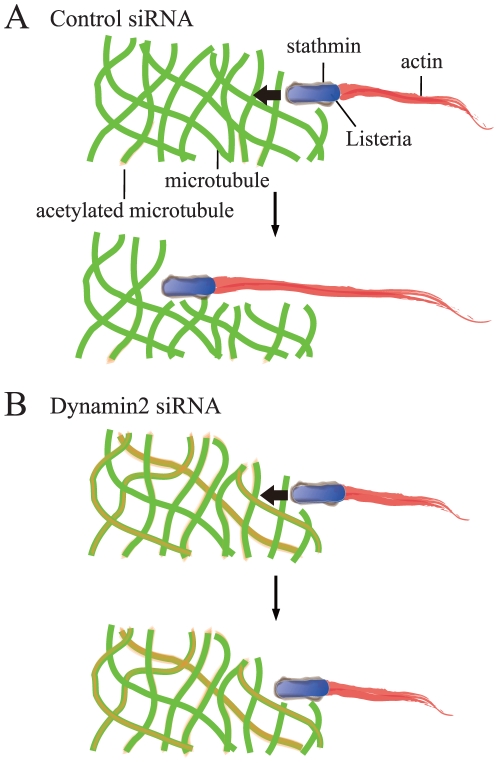
A model for actin comet action in dynamin2 siRNA-treated cells. *Listeria* recruits stathmin, which prevents microtubule polymerization, and then creates free space allowing the bacterium to move (A). In contrast, in dynamin2 siRNA-treated cells, the accumulation of stable microtubules creates a strong barrier to the bacterial pathogen, resulting in the reduction of both the length and speed of the actin comet (B).

As *Listeria* recruits stathmin, which inhibits microtubule polymerization or depolymerization [27], it is possible that the stathmin in dynamin2-depleted cells could not create free space to allow *Listeria* movement, and as a consequence, the speed of *Listeria* movement was decreased (a hypothetical model is illustrated in Fig. 6). Another possibility is that microtubules itself regulates actin polymerization by a signaling connection. Microtubule disassembly promotes the formation of actin stress fibers [28]. Drug, which disrupts microtubules, induces rapid assembly of actin filaments and focal adhesion [29], whereas microtubule stabilization with taxol attenuates these effects. Moreover, regulation of the actin filaments by microtubules requires Rho GTPases [30] and possibly dynamin [25]. It needs further investigation to uncover the molecular mechanism between the actin comet and microtubules, including the alteration of microtubule network and the signaling connections.

Previous reports show that several dynamin2 mutants, including K44A (dominant negative), D208N (reduced affinity for GTP), and ΔPRD (interaction with SH3-containing proteins, including cortactin) influence the morphology and motility of the actin comet [19], [20]. Of these, the first two mutants localize in the actin comet tail and their expression reduces the comet tail length. However, our present study showed that dynamin2 seems not to have a direct role on the actin filament of *Listeria*-induced actin comet. This implies that these mutants might induce abnormal actin rearrangement in the actin comet tail by overexpression of exogenous proteins. ΔPRD does not localize in the actin comet tail, but its expression reduces the comet tail length. One possibility is that PRD interacts with microtubules, and ΔPRD may affect the dynamic instability of microtubules, which indirectly influences on actin comet formation. Second possibility is that dynamin plays as an enzyme, which controls actin dynamics at actin comet, and dynamin2 directly regulates actin rearrangement in the actin comet or through other SH3-containing proteins. Interestingly, a recent report has shown that dynamin regulates actin polymerization through direct dynamin-actin interactions [25]. In this study, we could not exclude the possibility that dynamin regulates directly the actin polymerization. At least in part, however, microtubules contribute dynamin-dependent actin comet formation induced by *Listeria*. Based on the finding that cortactin also regulates actin polymerization and localizes on actin comet, but has no significant role on the length and the speed of actin comet, dynamin and/or cortactin may adjust actin bundle for efficient propulsion. Hence, further detailed analysis is required to fully understand the regulation of the actin comet by dynamin and cortactin.

## Materials and Methods

### Antibodies and DNA construction

Mouse antibody against cortactin was purchased from Millipore; goat antibody against dynamin2 (C-18) from Santa Cruz; rabbit antibody against V5 from Chemicon; and Alexa488-conjugated donkey anti-goat IgG, Alexa488 goat anti-mouse IgG, Alexa488 goat anti-rabbit IgG, Alexa555 human transferrin and rhodamine red-X-conjugated phalloidin from Invitrogen. Mouse antibody against α-tubulin, acetylated tubulin, colchicine and taxol were purchased from Sigma-Aldrich. pEGFP-actin was purchased from Clontech. pcDNA4-V5/His-dynamin2 WT and K44A was described previously [18].

### Cells, bacteria, and growth conditions

HeLa cells were maintained in DMEM containing 10% fetal bovine serum, penicillin/streptomycin, and fungizone at 37°C and 5% CO_2_. *Listeria* was a generous gift from K. Yokota (Okayama University, Japan). *Listeria* was grown at 37°C in brain–heart infusion medium (BHI; Difco Laboratories). To establish GFP-actin expressing HeLa cells, GFP-actin was introduced into the cells using Effectene (Qiagen), according to the manufacturer's instructions. Cells that stably expressed GFP-actin were selected using geneticin (Sigma).

### *Listeria* infection

*Listeria* was grown in BHI medium at 37°C overnight. HeLa cells were incubated in DMEM/10% FBS with no antibiotics. Untreated or siRNA-treated HeLa cells were infected with 1.0×10^7^/ml bacteria for 1 h. The cells were washed with PBS and incubated with fresh DMEM/10% FBS with no antibiotics at 37°C for 5 h. Taxol and colchicine was added to the medium, as required, at 50 µg/ml and 10 µM, respectively.

### Immunofluorescence

HeLa cells were fixed with 3.7% formaldehyde in PBS for 15 min at room temperature and washed three times with PBS. To perform the internalization assay, cells were incubated with 10 µg/ml Alexa555 transferrin at 37°C for 30 min prior to fixation. HeLa cells were treated with blocking buffer (1% BSA and 0.1% Triton X-100 in PBS) for 30 min and then incubated with primary antibodies for 45 min at room temperature. After three washes with PBS, HeLa cells were incubated for 30 min at room temperature with secondary antibodies. After another three washes with PBS, the cells were mounted in Prolong Gold Antifade Reagent (Invitrogen) and analyzed using a spinning disc confocal microscope system (CSU10; Yokogawa Electric Co.) on an inverted microscope (IX71; Olympus) equipped with an Ar/Kr laser. Images were acquired using an UPlan-Apochromat 100× NA 1.35 oil immersion objective (Olympus) and an electron-multiplying charge-coupled device camera (iXon; Andor Technology). Image capture and acquisition were performed using the MetaMorph software (MDS Analytical Technologies). Image analyses were performed using the ImageJ software (National Institutes of Health).

### RNAi

Human cortactin-specific siRNAs (cortactin siRNA 1, 5′-CCGAAUGGAUAAGUCAGCU-3′; cortactin siRNA 2, 5′-GGUUUCGGCGGCAAAUACG-3′), human dynamin2-specific siRNAs (dynamin2 siRNA 1, 5′-GGACUUACGACGGGAGAUC-3′; dynamin2 siRNA 2, 5′-GGAUAUUGAGGGCAAGAAG-3′), and negative control siRNA were purchased from Applied Biosystems. The siRNAs were introduced into HeLa cells using Lipofectamine 2000 (Invitrogen) according to the manufacturer's instructions. Immunoblot analysis was performed as previously described [18].

### Live imaging and data analysis

Live imaging was performed as described previously [31], [32]. The cells were incubated in culture medium at 37°C and 5% CO_2_ using a stage incubator (MI-IBC; Olympus) and digital gas mixer (GM-2000; Tokai Hit), and imaged using the aforementioned confocal system. The fluorescent signals were analyzed using MetaMorph and ImageJ. In brief, HeLa cells stably expressing GFP-actin were imaged at 5 s intervals 72 h after transfection with siRNAs. All data were analyzed for significance by Student's *t*-test.

## Supporting Information

Movie S1 **Dynamics of actin comet, induced by** ***Listeria*** **, visualized by GFP-actin.** HeLa cells stably expressing GFP-actin were transfected with control siRNA, infected with *Listeria*, and observed. Images were collected every 5 s for 300 s. Frame rate, 7 frames/s.
(MOV)

Movie S2 **The speed of** ***Listeria*** **movement decreased in dynamin2 siRNA cells.** HeLa cells stably expressing GFP-actin were transfected with dynamin2 siRNA, infected with *Listeria*, and observed. Images were collected every 5 s for 300 s. Frame rate, 7 frames/s.
(MOV)

Movie S3 **Actin comet, induced by** ***Listeria*** **, was elongated by taxol treatment.** HeLa cells stably expressing GFP-actin were transfected with control siRNA, infected with *Listeria*, treated with 50 µg/ml taxol, and observed. Images were collected every 5 s for 120 s. Frame rate, 7 frames/s.
(MOV)

Movie S4 **Actin comet in dynamin2 siRNA cells was elongated by taxol treatment.** HeLa cells stably expressing GFP-actin were transfected with dynamin2 siRNA, infected with *Listeria*, treated with 50 µg/ml taxol, and observed. Images were collected every 5 s for 105 s. Frame rate, 7 frames/s.
(MOV)
